# Hi-Plex for high-throughput mutation screening: application to the breast cancer susceptibility gene *PALB2*

**DOI:** 10.1186/1755-8794-6-48

**Published:** 2013-11-08

**Authors:** Tú Nguyen-Dumont, Zhi L Teo, Bernard J Pope, Fleur Hammet, Maryam Mahmoodi, Helen Tsimiklis, Nelly Sabbaghian, Marc Tischkowitz, William D Foulkes, Kathleen Cuningham Foundation Consortium for research into Familial Breast cancer (kConFab), Graham G Giles, John L Hopper, Australian Breast Cancer Family Registry, Melissa C Southey, Daniel J Park

**Affiliations:** 1 Genetic Epidemiology Laboratory, Department of Pathology, The University of Melbourne, Melbourne, VIC 3010, Australia; 2 Victorian Life Sciences Computation Initiative, 187 Grattan Street, Carlton, Melbourne, VIC 3010, Australia; 3 Department of Computing and Information Systems, The University of Melbourne, Melbourne, VIC 3010, Australia; 4 Jewish General Hospital, Lady Davis Institute, Montréal, QC H3T 1E2, Canada; 5 Department of Medical Genetics, University of Cambridge, Cambridge, UK; 6 Departments of Oncology and Human Genetics, McGill University, Montréal, QC H2W 1S6, Canada; 7 Centre for Molecular, Environmental, Genetic and Analytic Epidemiology, The University of Melbourne, Melbourne, VIC 3010, Australia; 8 Cancer Epidemiology Centre, The Cancer Council Victoria, Rathdowne Street, Carlton 3052, Australia

**Keywords:** Hi-Plex, Massively parallel sequencing, Mutation screening, *PALB2*, Molecular diagnostics

## Abstract

**Background:**

Massively parallel sequencing (MPS) has revolutionised biomedical research and offers enormous capacity for clinical application. We previously reported Hi-Plex, a streamlined highly-multiplexed PCR-MPS approach, allowing a given library to be sequenced with both the Ion Torrent and TruSeq chemistries. Comparable sequencing efficiency was achieved using material derived from lymphoblastoid cell lines and formalin-fixed paraffin-embedded tumour.

**Methods:**

Here, we report high-throughput application of Hi-Plex by performing blinded mutation screening of the coding regions of the breast cancer susceptibility gene *PALB2* on a set of 95 blood-derived DNA samples that had previously been screened using Sanger sequencing and high-resolution melting curve analysis (n = 90), or genotyped by Taqman probe-based assays (n = 5). Hi-Plex libraries were prepared simultaneously using relatively inexpensive, readily available reagents in a simple half-day protocol followed by MPS on a single MiSeq run.

**Results:**

We observed that 99.93% of amplicons were represented at ≥10X coverage. All 56 previously identified variant calls were detected and no false positive calls were assigned. Four additional variant calls were made and confirmed upon re-analysis of previous data or subsequent Sanger sequencing.

**Conclusions:**

These results support Hi-Plex as a powerful approach for rapid, cost-effective and accurate high-throughput mutation screening. They further demonstrate that Hi-Plex methods are suitable for and can meet the demands of high-throughput genetic testing in research and clinical settings.

## Background

Recently, there has been considerable discussion regarding how massively parallel sequencing (MPS) can optimally be applied in the context of clinical genetics services. Whole-genome MPS remains prohibitive in terms of cost, throughput, data handling and bioinformatic analysis complexity, as well as challenging clinical interpretation and raising many issues around the ethics of reporting results. Targeted MPS can address these issues by efficiently restricting clinical testing to sets of genes or genomic regions with known diagnostic value, while providing marked time- and cost-related advantages over traditional Sanger sequencing-based strategies.

We previously developed and reported Hi-Plex, a streamlined highly-multiplexed PCR approach for MPS library preparation, using DNA derived from both lymphoblastoid cell line and formalin-fixed, paraffin-embedded tumour tissue [1]. Our Hi-Plex library-building method integrates simple, automated primer design software that enables control of amplicon size. Importantly, this feature allows complete overlap of read pairs following paired-end sequencing to facilitate stringent downstream filtering of sequencing errors. We recently demonstrated that Hi-Plex using hybrid adapter primers (containing 5′-TruSeq compatible and 3′-Ion Torrent compatible sequences) can produce libraries suitable for both the Ion Torrent (PGM and Proton instruments, Life Technologies, Carlsbad, CA, USA) and TruSeq (MiSeq and HiSeq instruments, Illumina, San Diego, CA, USA) systems, which currently represent the two most commonly used MPS chemistries [2].

To assess the effectiveness of Hi-Plex in a high-throughput context, we used the MiSeq platform to perform mutation screening of 95 specimens, including three duplicated specimens, screened previously for genetic variants in the breast cancer susceptibility gene *PALB2* (GenBank reference sequence NM_024675; MIM#610355). Variant calling was blinded to the known *PALB2* germline status.

## Methods

### DNA samples

Our sample set consisted of 95 blood-derived DNAs derived from women affected by breast cancer that had been screened previously for mutations in the coding and flanking intronic regions of *PALB2* (n = 90) or genotyped for known *PALB2* pathogenic mutations (n = 5)*.* All participants provided written informed consent for participation in the study. This study was approved by The University of Melbourne Human Research Ethics Committee.

Biological samples were provided by the Australian Breast Cancer Family Registry (ABCFR, 91 specimens, of which three were duplicated specimens) and the Kathleen Cuningham Foundation Consortium for research into Familial Breast cancer (kConFab, Melbourne, Australia, four specimens). DNAs from both resources were extracted using QIAamp DNA Blood Kit (Qiagen, Hilden, Germany). Quant-iT™ PicoGreen® dsDNA Assay Kit (Life Technologies) was used for quantification.

Previous screens were done by Sanger sequencing and high-resolution melting curve analysis (HRM) for 85 specimens, including the duplicates, whereas HRM only was applied to five specimens. We included five specimens carrying pathogenic non-sense mutations identified previously by Taqman probe-based assays: *PALB2:*c.196C>T (n = 1) and *PALB2*:c.3113G>A (n = 4). Sanger sequencing was performed as previously described in [3] (unpublished data). HRM and Taqman probe-based assays are described in [4] and results of variant detection are reported in [4,5].

### Mutation screening using Hi-Plex

This Hi-Plex assay was designed to target the *PALB2* and *XRCC2* genes. However, genotyping aspects of this study focus on *PALB2* only, as we did not have a similar test set with genotype data for *XRCC2.*

Sixty primer pairs targeting the protein coding and some flanking intronic and untranslated regions of *PALB2* and *XRCC2* are described in [1] and Additional file 1. Dual-indexed hybrid adapter primer sets are described in Additional file 2. All oligonucleotides were obtained from Integrated DNA Technologies (Coralville, IA, USA).

96 individual PCR reactions (95 specimen DNAs and one no-template control) were performed in a standard skirted PCR plate, in a final volume of 50 μl, with1X Phusion® HF PCR buffer (ThermoScientific, Waltham, MA, USA), 2 units of Phusion Hot Start II High-Fidelity DNA Polymerase (ThermoScientific), 400 μM dNTPs (Bioline, London, UK), approximately 0.5 μM gene-specific primer pool (individual gene-specific primer concentrations vary and are described in [2]), 2.5 mM MgCl_2_ (ThermoScientific) and 25 ng input genomic DNA. The following steps were then applied to conduct PCR: 98°C for 1 min, 6 cycles of [98°C for 30 sec, 50°C for 1 min, 55°C for 1 min, 60°C for 1 min, 65°C for 1 min, 70°C for 1 min], addition of 2 μM each dual-indexed hybrid N50#_TSIT_A and N70#_TSIT_P adapter primers, then a further 19 cycles of [98°C for 30 sec, 50°C for 1 min, 55°C for 1 min, 60°C for 1 min, 65°C for 1 min, 70°C for 1 min], followed by incubation at 60°C for 20 min. Five μl of each reaction were pooled before subjecting the resulting barcoded library (including the 96 sub-libraries) to electrophoresis on a 2% HR-agarose gel (Life Technologies). Size selection, gel extraction and purification were performed as described previously [1].

The library was then sequenced on a MiSeq instrument, using the MiSeq Reagent kit v2 300 cycles (Illumina). Prior to performing the run, 3.4 μL of 100 μM sequencing primers were added to the respective read1, read2 and i7 primer reservoirs in the reagent cartridge. Sequencing primers were obtained from Integrated DNA Technologies (sequences are provided in Additional file 2).

Sequencing data were mapped to the entire human genome (hg19) using bowtie2-2.1.0 [6] applying default parameters except for --trim5 20 --trim3 20. Bedtools v2.16.1 [7] was used to compute on-target coverage. We used ROVER variant caller, a software tool developed in-house and made available at https://github.com/bjpop/rover to perform automated variant calling. To be called in this application, genetic variants had to appear in i) both members of read-pairs; ii) at least 2 read-pairs; and iii) ≥ 15% of read-pairs. Homozygous variants were called when the minor allele was present in ≥85% of read-pairs. The tool also reports the number of read pairs covering each targeted amplicon. Sequencing statistics reported in this paper (on-target and coverage calculations) include both *XRCC2* and *PALB2*, as they represent all the targeted regions. To assess the efficiency of the 60-plex assay across all 95 specimens, depth of coverage data were reported for 60 × 95 = 5,700 amplicons in total.

When validation was required for a genetic variant identified by Hi-Plex but not reported in previous screens, Sanger sequencing was performed using BigDye Terminator v3.1 (Life Technologies), according to the manufacturer’s instructions.

## Results and discussion

In our set of 95 samples, of reads mapping to the hg19 human genome build an average of 96.62% were on target. Across samples, the on-target rate ranged from 93.01% to 98.26% and the total number of reads that mapped on-target ranged from 7,933 to 171,466. When considering only correctly paired, on-target reads, we observed that 99.93% (5,696/5,700) of amplicons were represented at ≥10× coverage, across samples. Additionally, we found that 88.3% (5037/5700), 96.02% (5472/5700), 98.54% (5617/5700) and 99.30% (5660/5700) of amplicons were represented within 5-fold, 10-fold, 20-fold and 30-fold of the median coverage. Additional file 3 illustrates the coverage distribution across a sample of BAM files.

We accurately detected all 56 variant calls identified through previous mutation screening by Sanger sequencing and/or HRM, and Taqman probe-based genotyping. Heterozygous variants were observed in 37.23% (35/94) to 62.33% (513/823) of read-pairs (median = 51.23%). No false positive calls were assigned. All three pairs of duplicated samples yielded concordant genotypes.

The 56 calls comprised instances of 11 distinct genetic variants, including two non-sense variants (*PALB2:*c.196C>T and *PALB2:*c.3113G>A), two frameshift variants (*PALB2:*c.1947_1948insA and *PALB2:*c.2982_2983insT), four missense variants (*PALB2:*c.1010T>C, *PALB2:*c.1676A>G, *PALB2:*c.2014G>C and *PALB2:*c.2993G>A) and three synonymous variants (*PALB2:*c.1572A>G, *PALB2:*c.3300T>G and *PALB2:*c.3495G>A). Additional information regarding genotyping results is available in Table 1.

**Table 1:** ***PALB2 *** **variants identified in previous screens (Sanger sequencing and HRM) or genotyping assays (Taqman probe-based), and detected via Hi-Plex**

**Variant type**	**Nucleotide change**^**a**^	**Protein change**	**rs number**	**Number of carriers (detected by all used methods)**	**Number of carriers (detected by Hi-Plex only)**
Non-sense	c.196C>T	p.Gln66*	rs180177083	2 heterozygotes^b^	
	c.3113G>A	p.Trp1038*	rs180177132	4 heterozygotes^c^	
Frameshift	c.1947_1948insA	p.Glu650fs*13	-	1 heterozygote	
	c.2982_2983insT	p.Ala995fs*16	rs180177127	1 heterozygote	
Missense	c.1010T>C	p.Leu337Ser	rs45494092	5 heterozygotes^d^	
	c.1676A>G	p.Gln559Arg	rs152451	13 heterozygotes^d^	1 heterozygote^e,f^
				1 homozygote	
	c.2014G>C	p.Glu672Gln	rs45532440	8 heterozygotes	
				1 homozygote	
	c.2590C>T	p.Pro864Ser	rs45568339	-	1 heterozygote^e,g,h^
	c.2993G>A	p.Gly998Glu	rs45551636	7 heterozygotes	1 heterozygote^,e,f,g^
Synonymous	c.1470C>T	p.Pro490Pro	rs45612837	-	1 heterozygote^e,h,i^
	c.1572A>G	p.Ser524Ser	rs45472400	3 heterozygotes	
	c.3300T>G	p.Thr1100Thr	rs45516100	8 heterozygotes	
				1 homozygote	
	c.3495G>A	p.Ser1165Ser	-	1 heterozygote	

*indicates a protein truncation event.

^a^Number based on transcript sequence (NM_024675), +1 as A of ATG start codon.

^b^Including one sample that was genotyped by Taqman probe-based assay.

^c^All four samples were genotyped by Taqman probe-based assay.

^d^Including duplicated sample.

^e^Confirmed by Sanger sequencing.

^f^Previously screened by HRM only.

^g^Upon HRM curve re-analysis, the variant was apparent.

^h^Upon chromatogram re-analysis, the variant was apparent.

^i^Initially detected by HRM, not by Sanger sequencing.

Our screening by Hi-Plex also detected one *PALB2:*c.1470C>T carrier that was identified by HRM but not reported by prior Sanger sequencing, and one *PALB2:*c.2590C>T carrier that was not reported by either method. Upon re-analysis of the respective chromatograms and HRM curve, both variants were apparent in the expected samples (Additional file 4).

Discordant results were observed for two samples screened by Hi-Plex and HRM methods. The *PALB2:*c.2993G>A variant was detectable upon re-analysis of the HRM curve, whereas the *PALB2:*c.1676A>G carrier was not (Table 1). All four additionally identified variants were confirmed by follow-up Sanger sequencing.

Here, we have validated that Hi-Plex is capable of accurate, cost-effective and rapid high-throughput mutation screening using a series of 95 specimens previously characterized for *PALB2* genotype.

By performing single-step, highly-multiplexed PCR library-building, we avoided multiple manipulations, and waste of biological material and reagents associated with alternative methods [8]. Results reported here demonstrate that not only does Hi-Plex extensively reduce labour associated with amplification protocol optimization and library preparation, it also allows accurate screening without the need for normalisation of individual barcoded libraries before pooling and sequencing.

Easy and rapid library preparation did not compromise sequencing efficiency as shown by the 99.93% of amplicons represented at ≥10×. It did not impact on the sensitivity and specificity of variant detection either. All previously identified genetic variants were detected using our method. Furthermore, no false positive variants were called. Discordant calls as compared to previous screens proved to be genuine variants following confirmatory Sanger sequencing or detectable upon re-analysis of chromatograms and/or HRM curves. As stated previously, Hi-Plex’s experimental strategy includes a primer design tool that allows generation of primers for amplicons of a defined size, which should be shorter than the length of a sequencing read. As such, completely-overlapping reads can be achieved when performing paired-end sequencing. This allows stringent filtering of sequencing chemistry-induced artefacts by only considering variants that appear in both reads of pairs. In turn, this allows highly accurate variant detection.

The screen for genetic variations across 95 specimens reported here was achieved in two days at a cost of ~ AU$20/specimen, accounting for all aspects of library-building, MPS and analysis (including technician time). The equivalent Sanger sequencing-based screen would take approximately two weeks and confer a total cost of ~ AU$400/specimen.

This report shows that our Hi-Plex approach performs with a sensitivity and accuracy suitable for diagnostic application, while being more time- and cost-effective than Sanger sequencing, the current “gold standard” screening method. The mechanisms underlying Hi-Plex suggest that higher parallelization should be achievable without extensive protocol adjustment. Future experiments will involve increasing the level of multiplexing of Hi-Plex, with the aim of achieving robust thousands-plex multiplexing. Cost-effective and rapid methods for screening are highly desirable for mutation scanning, particularly in clinical settings, where eligibility is partly dictated by cost of testing. Lower screening costs could help facilitate the shift from single-gene to gene-panel screening and support a new approach to personalised clinical genetics service delivery.

## Conclusions

In the context of research and ‘gene association’ studies, Hi-Plex enables large-scale sequencing in genetic epidemiological studies at relatively low cost, with more flexibility than currently offered commercial solutions where targeted sequencing is often constrained to specific platforms. The latter confer design inflexibilities and are costly to re-design in a setting where screening strategies are often re-directed by recent findings. Hi-Plex’s intrinsic modular flexibility in terms of target region design, as well as sequencing platform, renders the approach highly attractive for an extensive range of clinical and research applications.

## Abbreviations

MPS: Massively parallel sequencing; HRM: High-resolution melting curve analysis; ABCFR: Australian Breast Cancer Family Registry; kConFab: Kathleen Cuningham Foundation Consortium for research into Familial Breast cancer.

## Competing interests

The authors declare that they have no competing interests.

## Authors’ contributions

TN-D, FH, MM and HT carried out the Hi-Plex experiments; ZLT carried out the HRM screening and Taqman genotyping assays; NS, MT and WDF contributed Sanger screening data; BJP wrote ROVER; TN-D carried out the bioinformatics analyses; TN-D and FH carried out the performance comparison between screening methods; kConFab, the ABCFR, GGG and JLH contributed research resources; MCS participated in the study design and coordination; DJP conceived the study, and participated in its design and coordination. All authors helped to draft the manuscript. All authors read and approved the final manuscript.

## Pre-publication history

The pre-publication history for this paper can be accessed here:

http://www.biomedcentral.com/1755-8794/6/48/prepub

## Supplementary Material

Additional file 1 **Hi-Plex primers used in this study.** The data provided correspond to the oligonucleotide sequences of all gene-specific primers used in this study. Click here for file

Additional file 2 **Dual-indexed hybrid adapters and MiSeq primers used in this study.** The data provided correspond to the oligonucleotide sequences of all adapters and sequencing primers used in this study. Click here for file

Additional file 3 **BAM files visualized using the Integrated Genome Viewer (IGV).** Alignment and coverage tracks for five randomly selected sample, following library preparation using Hi-Plex. The data provided correspond to the IGV view from 5 randomly selected BAM files. Click here for file

Additional file 4 **Chromatograms from *PALB2*:c.2014G>C and *PALB2*:c.2590C>T carriers (initial Sanger sequencing screening).** Hi-Plex identified one *PALB2*:c.2014G>C and one *PALB2*:c.2590C>T variant carriers, which were not reported in the previous Sanger sequencing screen. Both variants were detectable upon re-analysis of the initial chromatograms (A and B, respectively). The variant positions are indicated by an arrow. Genotypes are indicated on the figure. Click here for file
