# Supplementary material for: Hi-Plex for high-throughput mutation screening: application to the breast cancer susceptibility gene PALB2
Source: BMC Med Genomics. 2013 Nov 8;6:48. doi: 10.1186/1755-8794-6-48 (PMC3829211; doi:10.1186/1755-8794-6-48)
Supplement: Additional file 1 — Hi-Plex primers used in this study. The data provided correspond to the oligonucleotide sequences of all gene-specific primers used in this study. [file 1755-8794-6-48-S1.doc]

Additional Table 1: Hi-Plex primers used in this study. Lower case sequence text relates to adapter sequence regions. Upper case sequence text indicates gene-specific sequence regions.

| **Primer** | **5-prime to 3-prime sequence** |
| --- | --- |
| itPALB2_X1_F1 | ctctctatgggcagtcggtgattCTAAAACCCTGGGAAAGCGGG |
| itPALB2_X1_R1 | ctgcgtgtctccgactcagCGACGGCTGCTCTTTTCGTTC |
| itPALB2_X1_F2 | ctctctatgggcagtcggtgattTCGTCCATCGGGCAGGCGACA |
| itPALB2_X1_R2 | ctgcgtgtctccgactcagTCGCGCACTGAGGGTGCGATC |
| itPALB2_X1_F3 | ctctctatgggcagtcggtgattAGGAAGGAATGGGGAGCCCGG |
| itPALB2_X1_R3 | ctgcgtgtctccgactcagGCTCCACTGCTCGGCCGTCTA |
| itPALB2_X2_F1 | ctctctatgggcagtcggtgattACTGTTTTTAAATTGTTTGTACTATAA |
| itPALB2_X2_R1 | ctgcgtgtctccgactcagTGCCCAGTATTGTTGGTGTTTTT |
| itPALB2_X3_F1 | ctctctatgggcagtcggtgattAAATGAATAATAAAGCAGGCATAAG |
| itPALB2_X3_R1 | ctgcgtgtctccgactcagCGTGCCCAAAGAGCTGAAAAG |
| itPALB2_X3_F2 | ctctctatgggcagtcggtgattACTGTTTTCTTAATAGAATGCTTAAT |
| itPALB2_X3_R2 | ctgcgtgtctccgactcagTATTCTCAAATTAAGGTGTTATAGTA |
| itPALB2_X4_F1 | ctctctatgggcagtcggtgattATCATCATCATCATCATCATCAAA |
| itPALB2_X4_R1 | ctgcgtgtctccgactcagATTGTGAACCACTTTTGCCAACT |
| itPALB2_X4_F2 | ctctctatgggcagtcggtgattTGTTAACAATCGACAGGCTAGA |
| itPALB2_X4_R2 | ctgcgtgtctccgactcagCCACTGAAGATAATGACTTGTC |
| itPALB2_X4_F3 | ctctctatgggcagtcggtgattGCTTGGGCAACTGCCTTCCTA |
| itPALB2_X4_R3 | ctgcgtgtctccgactcagCCAAAGTGAAATTAGGATGTCTG |
| itPALB2_X4_F4 | ctctctatgggcagtcggtgattTTGGTTGTCCTGTGCATGTGC |
| itPALB2_X4_R4 | ctgcgtgtctccgactcagGGCTGTCATTCAGAGTCATTTG |
| itPALB2_X4_F5 | ctctctatgggcagtcggtgattTTTTAAACCCTTTTTTCTTGACATC |
| itPALB2_X4_R5 | ctgcgtgtctccgactcagAACATTCTTGCACAGTGCCTGA |
| itPALB2_X4_F6 | ctctctatgggcagtcggtgattTCTGCAGGAAACAGAAGGCCT |
| itPALB2_X4_R6 | ctgcgtgtctccgactcagACACTCTTGATGGCAGGAATG |
| itPALB2_X4_F7 | ctctctatgggcagtcggtgattGAATCTCACTTTCCTGAAGATTTT |
| itPALB2_X4_R7 | ctgcgtgtctccgactcagCATGTTCTCTAAATGAACTCACC |
| itPALB2_X4_F8 | ctctctatgggcagtcggtgattTTTCATTTGCTGGTAAGTTATTGTA |
| itPALB2_X4_R8 | ctgcgtgtctccgactcagGGCAAAAAAATGACTGTCTCTAC |
| itPALB2_X4_F9 | ctctctatgggcagtcggtgattGCTTTATTTACAAGGAGGTTATCT |
| itPALB2_X4_R9 | ctgcgtgtctccgactcagAGCACCTTGAACACATTCCTC |
| itPALB2_X4_F10 | ctctctatgggcagtcggtgattAGTAAGTTCACTGCTACCTTTAG |
| itPALB2_X4_R10 | ctgcgtgtctccgactcagTGCCCAACCAGAAAAAGGTGTT |
| itPALB2_X4_F11 | ctctctatgggcagtcggtgattATTAGGTCTTCTTAGGAATGTATC |
| itPALB2_X4_R11 | ctgcgtgtctccgactcagTAACTGAAATAAGAACTCACCTTTT |
| itPALB2_X4_F12 | ctctctatgggcagtcggtgattTCTGGAAGTTCAGATTTAAGACTT |
| itPALB2_X4_R12 | ctgcgtgtctccgactcagGAGACTGTGTCTTTGGCACTG |
| itPALB2_X4_F13 | ctctctatgggcagtcggtgattTTTCCCAGACAATCTGAGTGAAT |
| itPALB2_X4_R13 | ctgcgtgtctccgactcagCATTTTCCCCACAGGGTCAGT |
| itPALB2_X4_F14 | ctctctatgggcagtcggtgattCTTTTGCTCACCACTAGGGTC |
| itPALB2_X4_R14 | ctgcgtgtctccgactcagGGAGAAAAGACATCTATCACACT |
| itPALB2_X4_F15 | ctctctatgggcagtcggtgattAAGGACTCAGGCCCAACATCA |
| itPALB2_X4_R15 | ctgcgtgtctccgactcagATAAGAAAAATATAAGTTATATACATTTT |
| itPALB2_X5_F1 | ctctctatgggcagtcggtgattACTAAGGCATTTCATTCCTTCAG |
| itPALB2_X5_R1 | ctgcgtgtctccgactcagAACACCTCCACCCATTGAGTC |
| itPALB2_X5_F2 | ctctctatgggcagtcggtgattGAGCTGATTTTCTTTAAAAGTGAAT |
| itPALB2_X5_R2 | ctgcgtgtctccgactcagAATTCGACAGTTCAGGCAGCC |
| itPALB2_X5_F3 | ctctctatgggcagtcggtgattGGGTGGTATGTGGTTTTGCTG |
| itPALB2_X5_R3 | ctgcgtgtctccgactcagGCTCCTATGAAAAAGCATCTACA |
| itPALB2_X5_F4 | ctctctatgggcagtcggtgattGCAAGTTCGTCCAGCAACTTC |
| itPALB2_X5_R4 | ctgcgtgtctccgactcagTAAATACGGTTGCGCCTGATGA |
| itPALB2_X5_F5 | ctctctatgggcagtcggtgattTCTGTGGTAGGCCTGTCATTA |
| itPALB2_X5_R5 | ctgcgtgtctccgactcagACCTTATTGTTCTACCAGGAAAAT |
| itPALB2_X5_F6 | ctctctatgggcagtcggtgattAGTTTGGCCTTTTGGGATGTG |
| itPALB2_X5_R6 | ctgcgtgtctccgactcagAAAATGTTTGGAGAGAGACATCTT |
| itPALB2_X5_F7 | ctctctatgggcagtcggtgattGGAAAAATACAGCTTCCCTCTTT |
| itPALB2_X5_R7 | ctgcgtgtctccgactcagAGTATCACAGACTTTCAGTTACC |
| itPALB2_X5_F8 | ctctctatgggcagtcggtgattTAAGAGGTCCAAAGTCTTCATCA |
| itPALB2_X5_R8 | ctgcgtgtctccgactcagTCTTGGAGTAATAGTGCTTATTTAT |
| itPALB2_X5_F9 | ctctctatgggcagtcggtgattGTGAAAGCATCATCATCCAAGG |
| itPALB2_X5_R9 | ctgcgtgtctccgactcagGTGAAACAGATTGTCTGTTTTGTT |
| itPALB2_X6_F1 | ctctctatgggcagtcggtgattACACGAGACACTGGAAGAGAAT |
| itPALB2_X6_R1 | ctgcgtgtctccgactcagTACATAAAGTGTAGACTAATGATGT |
| itPALB2_X7_F1 | ctctctatgggcagtcggtgattATTATCAGGCAAATGGCTGCAAA |
| itPALB2_X7_R1 | ctgcgtgtctccgactcagTGTATCATAACTGCTTGCGAAGA |
| itPALB2_X7_F2 | ctctctatgggcagtcggtgattGAGCTTTCCAAAGAGAAACTACA |
| itPALB2_X7_R2 | ctgcgtgtctccgactcagCACTTTAACAGAACTGTTGCCAT |
| itPALB2_X8_F1 | ctctctatgggcagtcggtgattAACAAATCTCTCTCTCTTTAGATC |
| itPALB2_X8_R1 | ctgcgtgtctccgactcagGAAATCAGAGAGATCAGGTATGT |
| itPALB2_X8_F2 | ctctctatgggcagtcggtgattAAAACAAATCACTCCTTGGGAATT |
| itPALB2_X8_R2 | ctgcgtgtctccgactcagAGCATAATTTTTGGCTGCTTTGTT |
| itPALB2_X9_F1 | ctctctatgggcagtcggtgattTGATAAAATCATTCTTCATCTAATAG |
| itPALB2_X9_R1 | ctgcgtgtctccgactcagCTGTGCTTGGCCTGACAAAGA |
| itPALB2_X9_F2 | ctctctatgggcagtcggtgattCACTGCTACTAACTAGCCTCC |
| itPALB2_X9_R2 | ctgcgtgtctccgactcagCACATCACCCCATTTTTCCTTAT |
| itPALB2_X10_F1 | ctctctatgggcagtcggtgattTAAAATTAGAGGTATATCCTCATAC |
| itPALB2_X10_R1 | ctgcgtgtctccgactcagGGAGACTATACTAACTTTTGCTG |
| itPALB2_X10_F2 | ctctctatgggcagtcggtgattCTTCTTGCATCCCTTGGACCT |
| itPALB2_X10_R2 | ctgcgtgtctccgactcagGAGAAGGGCTACCTAGAGACT |
| itPALB2_X11_F1 | ctctctatgggcagtcggtgattCATTGCTATCCAATTTTGAAAAAAG |
| itPALB2_X11_R1 | ctgcgtgtctccgactcagCACAAAGCCTATTCTGAAATGGT |
| itPALB2_X11_F2 | ctctctatgggcagtcggtgattGGTCCCAGCCAGTCATTACTT |
| itPALB2_X11_R2 | ctgcgtgtctccgactcagAGAATGTGATCAGCTTATTTATTTTT |
| itPALB2_X12_F1 | ctctctatgggcagtcggtgattTGCACAGTGCCTTTCAGAATGT |
| itPALB2_X12_R1 | ctgcgtgtctccgactcagCCTGTGTTTCAGCTCATTGTGA |
| itPALB2_X12_F2 | ctctctatgggcagtcggtgattCTGAGAGTCGTCTTAGGGTTAA |
| itPALB2_X12_R2 | ctgcgtgtctccgactcagCTTGACAGTCTATTTGGGATATTT |
| itPALB2_X13_F1 | ctctctatgggcagtcggtgattATTGATTAAATATCCTGGTTGTATAA |
| itPALB2_X13_R1 | ctgcgtgtctccgactcagCATGTGCTTTTAAAGGTGTACATA |
| itPALB2_X13_F2 | ctctctatgggcagtcggtgattTAAATATTTATTGCCATTTGAAGCTT |
| itPALB2_X13_R2 | ctgcgtgtctccgactcagTATTCTAATGATGGTAGCACCAC |
| itPALB2_X13_F3 | ctctctatgggcagtcggtgattATACATAAATGTACATCCAAGATCA |
| itPALB2_X13_R3 | ctgcgtgtctccgactcagTATTGGGCCTCTTAGTATTTTTTG |
| itPALB2_X13_F4 | ctctctatgggcagtcggtgattGATATTCTCCTTTATATTTAAAACTC |
| itPALB2_X13_R4 | ctgcgtgtctccgactcagTACAGACTCTCATTTGCTGGCT |
| itPALB2_X13_F5 | ctctctatgggcagtcggtgattCAAATATATTTCCATCTTTTTGTCC |
| itPALB2_X13_R5 | ctgcgtgtctccgactcagACTTCTGGAACAATTGCCATTTG |
| itPALB2_X13_F6 | ctctctatgggcagtcggtgattCACTGACCGAGAAGTAAGTCC |
| itPALB2_X13_R6 | ctgcgtgtctccgactcagTTTTTTTAATTGTTTTTTGGATATGTAA |
| itXRCC2_X1_F1 | ctctctatgggcagtcggtgattGCCTTGTTCCCATCTCCCTCA |
| itXRCC2_X1_R1 | ctgcgtgtctccgactcagTTGGTGAATGGCGTTGGTGGC |
| itXRCC2_X1_F2 | ctctctatgggcagtcggtgattCAGGAGAGACTCAACTTTCCC |
| itXRCC2_X1_R2 | ctgcgtgtctccgactcagGCACACCCTATTGCGCATGCT |
| itXRCC2_X2_F1 | ctctctatgggcagtcggtgattATTTGCATTTATTTATATAAAGGTTGT |
| itXRCC2_X2_R1 | ctgcgtgtctccgactcagATAATGAGTTTTCCTTCTCTCTTC |
| itXRCC2_X3_F1 | ctctctatgggcagtcggtgattGAAAAATTTTAAGGCTTGCGTAGT |
| itXRCC2_X3_R1 | ctgcgtgtctccgactcagCAACCAATTTTCATTAGTTTCACG |
| itXRCC2_X3_F2 | ctctctatgggcagtcggtgattTTTTTTTAAACTGTTACTTTTTAAACAA |
| itXRCC2_X3_R2 | ctgcgtgtctccgactcagGATGTGGACATAGACTACAGAC |
| itXRCC2_X3_F3 | ctctctatgggcagtcggtgattTGCCATGCCTTACAGAGATAAG |
| itXRCC2_X3_R3 | ctgcgtgtctccgactcagTAAATGACTATCGCCTGGTTCTT |
| itXRCC2_X3_F4 | ctctctatgggcagtcggtgattCATTATAGTTTGTGTCGTTGCAAA |
| itXRCC2_X3_R4 | ctgcgtgtctccgactcagCTGTCAGCTTTTTACTGGATAGA |
| itXRCC2_X3_F5 | ctctctatgggcagtcggtgattCTTTCTCCTCCATTGACGCGG |
| itXRCC2_X3_R5 | ctgcgtgtctccgactcagTGTACTGCAGTAGTAGCACCC |
| itXRCC2_X3_F6 | ctctctatgggcagtcggtgattGTGAGTAAAGTGTAAGAAGTAAGT |
| itXRCC2_X3_R6 | ctgcgtgtctccgactcagTTACCACTTTGATATGCTCCGG |
| itXRCC2_X3_F7 | ctctctatgggcagtcggtgattCTGTGCTCAAGAATTGTAACTAG |
| itXRCC2_X3_R7 | ctgcgtgtctccgactcagGGAACAGGAAAAACAGAAATGCT |
| itXRCC2_X3_F8 | ctctctatgggcagtcggtgattTACATCGTGCTGTTAGGTGATAA |
| itXRCC2_X3_R8 | ctgcgtgtctccgactcagTGCAGACTTTGCATTTTGTAACC |
