# Supplementary material for: Hi-Plex for high-throughput mutation screening: application to the breast cancer susceptibility gene PALB2
Source: BMC Med Genomics. 2013 Nov 8;6:48. doi: 10.1186/1755-8794-6-48 (PMC3829211; doi:10.1186/1755-8794-6-48)
Supplement: Additional file 2 — Dual-indexed hybrid adapters and MiSeq primers used in this study. The data provided correspond to the oligonucleotide sequences of all adapters and sequencing primers used in this study. [file 1755-8794-6-48-S2.doc]

Additional Table 2: Dual-indexed hybrid adapters and MiSeq primers used in this study. For adapters, upper case sequence text relates to TruSeq sequences, underlined sequence text relates to Nextera-dual indices and lower case relates to Ion Torrent sequences.

| **Primer** | **5-prime to 3-prime sequence** |
| --- | --- |
| N501_TSIT_A | AATGATACGGCGACCACCGAGATCTACACTAGATCGCccatctcatccctgcgtgtctccgactcag |
| N502_TSIT_A | AATGATACGGCGACCACCGAGATCTACACCTCTCTATccatctcatccctgcgtgtctccgactcag |
| N503_TSIT_A | AATGATACGGCGACCACCGAGATCTACACTATCCTCTccatctcatccctgcgtgtctccgactcag |
| N504_TSIT_A | AATGATACGGCGACCACCGAGATCTACACAGAGTAGAccatctcatccctgcgtgtctccgactcag |
| N505_TSIT_A | AATGATACGGCGACCACCGAGATCTACACGTAAGGAGccatctcatccctgcgtgtctccgactcag |
| N506_TSIT_A | AATGATACGGCGACCACCGAGATCTACACACTGCATAccatctcatccctgcgtgtctccgactcag |
| N507_TSIT_A | AATGATACGGCGACCACCGAGATCTACACAAGGAGTAccatctcatccctgcgtgtctccgactcag |
| N508_TSIT_A | AATGATACGGCGACCACCGAGATCTACACCTAAGCCTccatctcatccctgcgtgtctccgactcag |
| N701_TSIT_P | CAAGCAGAAGACGGCATACGAGATTCGCCTTActccgctttcctctctatgggcagtcggtgat |
| N702_TSIT_P | CAAGCAGAAGACGGCATACGAGATCTAGTACGctccgctttcctctctatgggcagtcggtgat |
| N703_TSIT_P | CAAGCAGAAGACGGCATACGAGATTTCTGCCTctccgctttcctctctatgggcagtcggtgat |
| N704_TSIT_P | CAAGCAGAAGACGGCATACGAGATGCTCAGGActccgctttcctctctatgggcagtcggtgat |
| N705_TSIT_P | CAAGCAGAAGACGGCATACGAGATAGGAGTCCctccgctttcctctctatgggcagtcggtgat |
| N706_TSIT_P | CAAGCAGAAGACGGCATACGAGATCATGCCTActccgctttcctctctatgggcagtcggtgat |
| N707_TSIT_P | CAAGCAGAAGACGGCATACGAGATGTAGAGAGctccgctttcctctctatgggcagtcggtgat |
| N708_TSIT_P | CAAGCAGAAGACGGCATACGAGATCCTCTCTGctccgctttcctctctatgggcagtcggtgat |
| N709_TSIT_P | CAAGCAGAAGACGGCATACGAGATAGCGTAGCctccgctttcctctctatgggcagtcggtgat |
| N710_TSIT_P | CAAGCAGAAGACGGCATACGAGATCAGCCTCGctccgctttcctctctatgggcagtcggtgat |
| N711_TSIT_P | CAAGCAGAAGACGGCATACGAGATTGCCTCTTctccgctttcctctctatgggcagtcggtgat |
| N712_TSIT_P | CAAGCAGAAGACGGCATACGAGATTCCTCTACctccgctttcctctctatgggcagtcggtgat |
| TSIT_Read1 | CCATCTCATCCCTGCGTGTCTCCGACTCAG |
| TSIT_Read2 | CTCCGCTTTCCTCTCTATGGGCAGTCGGTGATT |
| TSIT_i7_read | AATCACCGACTGCCCATAGAGAGGAAAGCGGAG |
