# Supplementary figures and images for: Hi-Plex for high-throughput mutation screening: application to the breast cancer susceptibility gene PALB2
Source: BMC Med Genomics. 2013 Nov 8;6:48. doi: 10.1186/1755-8794-6-48 (PMC3829211; doi:10.1186/1755-8794-6-48)

## Slide 1
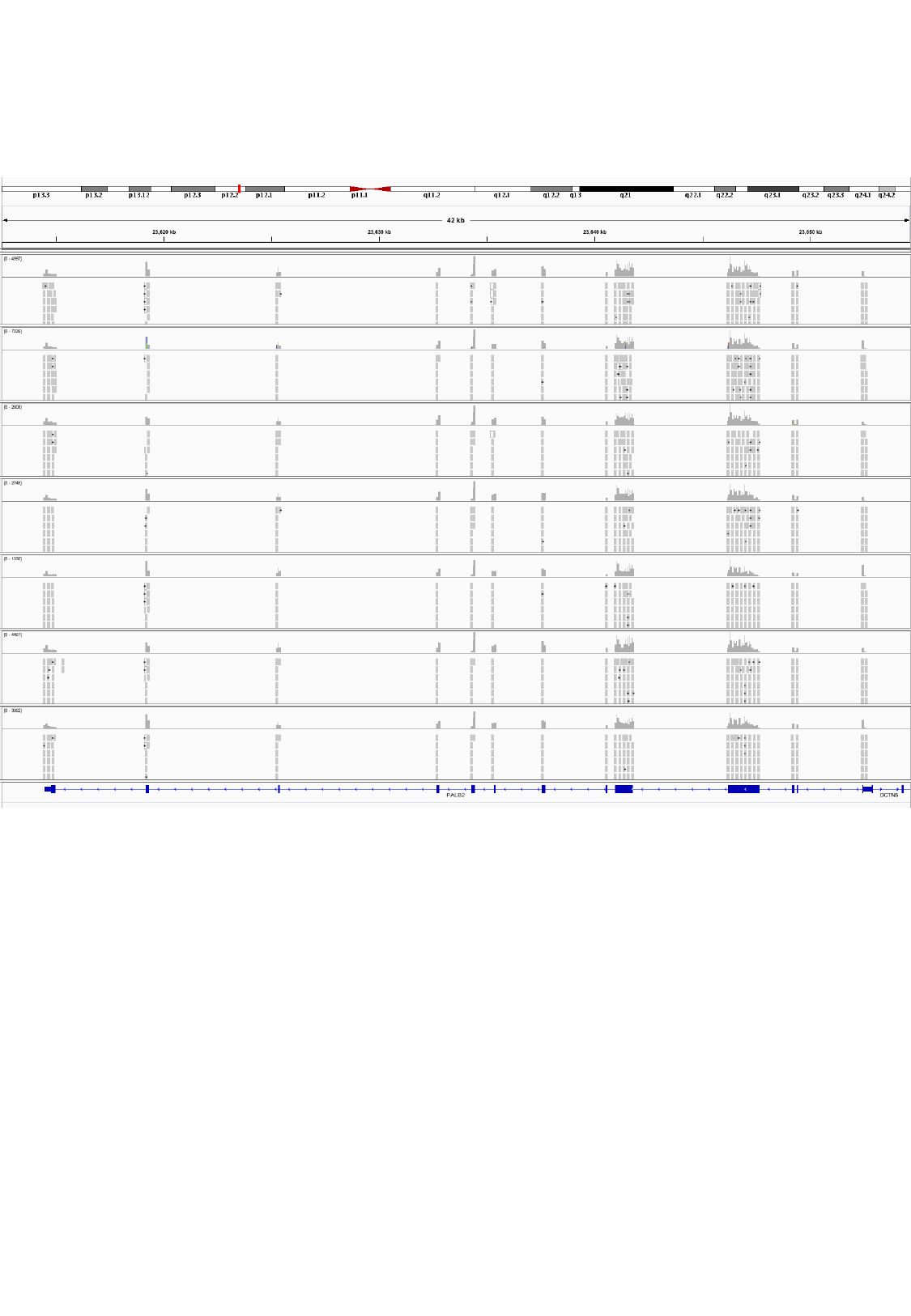

Supplement: Additional file 3 — BAM files visualized using the Integrated Genome Viewer (IGV). Alignment and coverage tracks for five randomly selected sample, following library preparation using Hi-Plex. The data provided correspond to the IGV view from 5 randomly selected BAM files. [file 1755-8794-6-48-S3.ppt]

## Slide 1
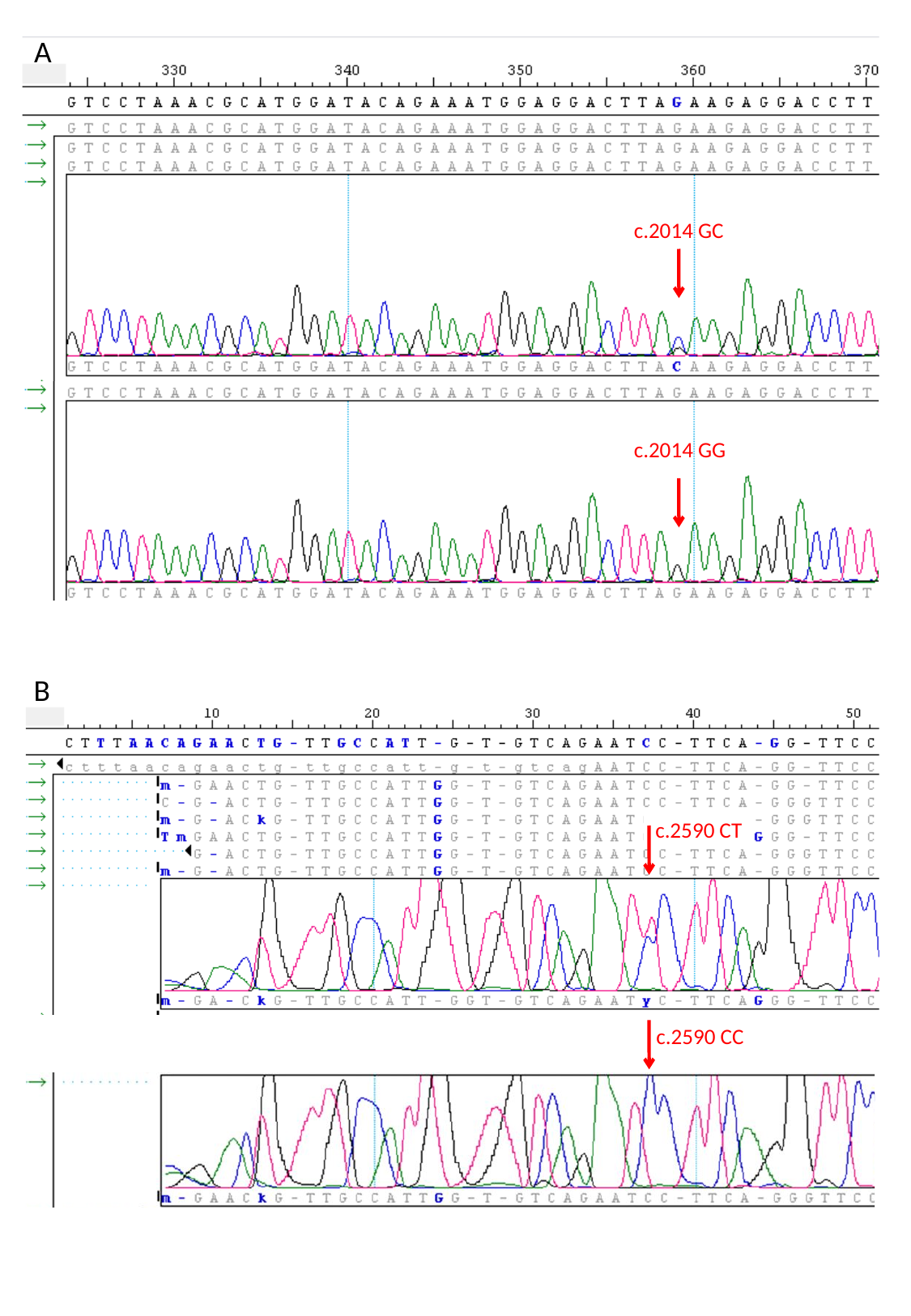

A
c.2014 GC
c.2014 GG
B
c.2590 CT
c.2590 CC

Supplement: Additional file 4 — Chromatograms from PALB2 :c.2014G>C and PALB2 :c.2590C>T carriers (initial Sanger sequencing screening). Hi-Plex identified one PALB2:c.2014G>C and one PALB2:c.2590C>T variant carriers, which were not reported in the previous Sanger sequencing screen. Both variants were detectable upon re-analysis of the initial chromatograms (A and B, respectively). The variant positions are indicated by an arrow. Genotypes are indicated on the figure. [file 1755-8794-6-48-S4.ppt]
